# Atypical Feeding and Drainage of Dural AV Fistula

**DOI:** 10.5334/jbr-btr.815

**Published:** 2016-03-07

**Authors:** Mehmet Sirik, Bulent Petik, Deniz Colak, Sukru Mehmet Erturk, Mehmet Karatas

**Affiliations:** 1 Adiyaman University School of Medicine, Department of Radiology, TR; 2 Adiyaman University School of Medicine, Department of Otolaryngology-Head and Neck Surgery, TR

**Keywords:** Dural AV fistula, Doppler ultrasonography, Multidetector computerized tomography (MDCT) angiography

## Abstract

Dural arteriovenous fistulas (DAVF) are frequent causes of pulsatile tinnitus. Color Doppler sonography may play a useful, complementary role to CTA/MRA and digital subtraction angiography (DSA) in the assessment of these anomalies’ characteristics, such as an ipsilateral increased flow volume and a low resistive index. In this article, we report a case of DAVF first detected with Color Doppler sonography that displayed an uncharacteristic venous drainage pattern.

## Introduction

Tinnitus is defined as the perception of sound when no external stimuli are present; it can be pulsatile or non-pulsatile [1]. Whereas tinnitus, in general, can be the only symptom of an underlying pathology, it can also be one of several symptoms of a neurological disorder. Pulsatile tinnitus, in particular, can be of vascular origin [1,2].

Dural arteriovenous fistulas (DAVF) are a heterogeneous collection of conditions that share arteriovenous shunts from dural vessels. Atherosclerosis, aneurysms or dissection of the internal carotid artery, cerebral sinus thrombosis, and some other rare pathologies are among the vascular causes of pulsatile tinnitus [1]. Because of the risk of development of various neurological symptoms, intracranial hypertension, hemorrhage, and even death, DAVFs need to be correctly diagnosed and characterized [1,2,3,4,5,6].

Carotid Doppler sonography (CDS) can be used as an efficient tool for screening patients with DAVF [2]. Findings that include reduced flow resistance, increased flow volume, increased systolic velocity, and increased diastolic velocity have been proposed to diagnose DAVFs [2].

We herein present a unique case with pulsatile tinnitus and associated abnormal CDS, which resulted from a DAVF, fed by the occipital artery and with extracranial drainage to the right brachiocephalic vein, in addition to the typical venous drainage to the transverse sinus. To the best of our knowledge, a drainage pattern like this has not been published before and is not included in the classification of DAVFs [7,8,9,10].

## Case Report

**Figure 1:**
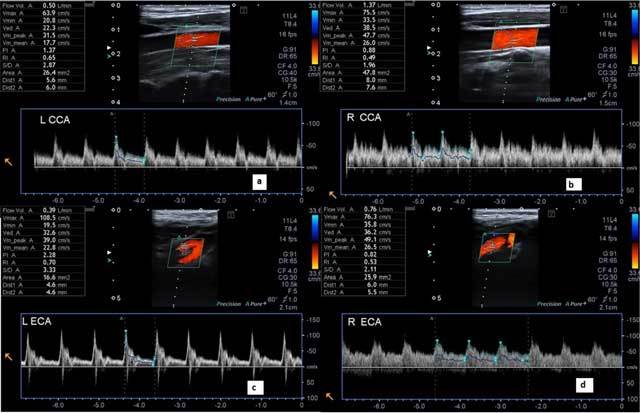
The peak systolic and end diastolic velocities, flow volume rates, and resistive indices of right (a) and left (b) carotid arteries and right (c) and left (d) external carotid arteries were determined with the carotid Doppler sonography.

A 39-year-old man presented a one-year history of pulsatile tinnitus in the right ear without a previous history of trauma, surgery, or any other disease. His ear, nose, and throat (ENT) examination and laboratory tests were normal. The patient was referred to our department for carotid Doppler sonography to exclude vascular causes of pulsatile tinnitus. B-mode CDS revealed normal carotid intima-media thickness and no plaque formation. Doppler mode demonstrated increased peak systolic and end diastolic velocities and a decreased resistive index (RI) of the right common carotid artery (75 cm/sec, 33 cm/sec, and 0.49, respectively) compared to the left common carotid artery (63 cm/sec, 20 cm/sec, and 0.65, respectively). The blood flow volume (1370 ml/min) of the right common carotid artery (CCA) was substantially higher than that of the left CCA (500 ml/min). Likewise, the blood flow volume (760 ml/min) of the right external carotid artery (ECA) was higher than that of the left ECA (390 ml/min), and the resistive index of the right ECA (0.53) was lower than that of the left ECA (0.70) (Figure 1).

**Figure 2:**
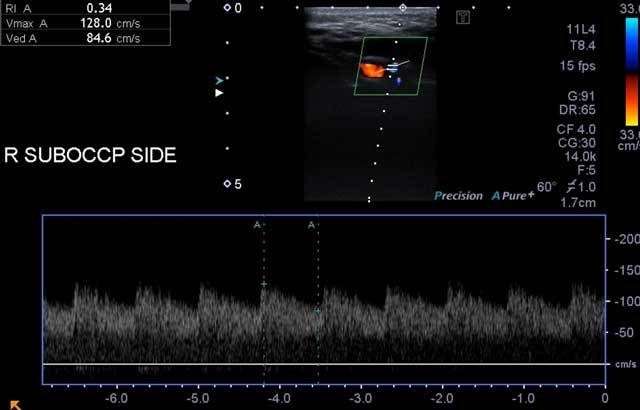
A vascular structure in the right suboccipital region showing high-speed, low-resistance blood flow pattern was confirmed with carotid Doppler sonography.

**Figure 3:**
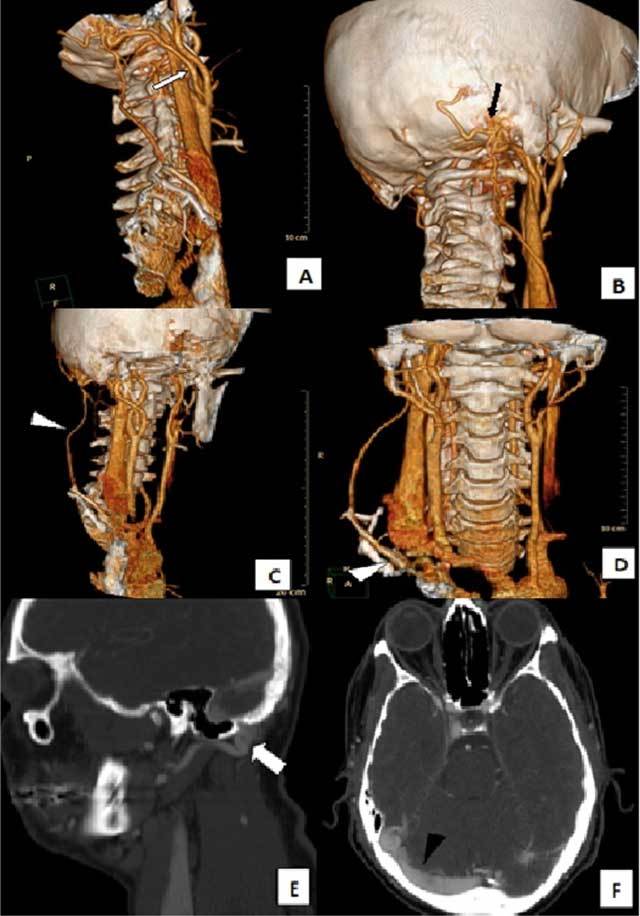
On CT angiography, (a) an enlarged right occipital artery was identified as the feeding artery of the DAVF (white arrow) and the (b) large, tortuous vascular structures were seen in the right mastoid region (black arrow). The (c–d) auxiliary/alternative venous drainage path to the right brachiocephalic vein (white arrowhead), (e) the location of the fistula in the suboccipital-mastoid region and enlarged vascular structures (thick white arrow), and (f) the typical drainage of DAVF to the right transverse-sigmoid sinus were determined.

**Figure 4:**
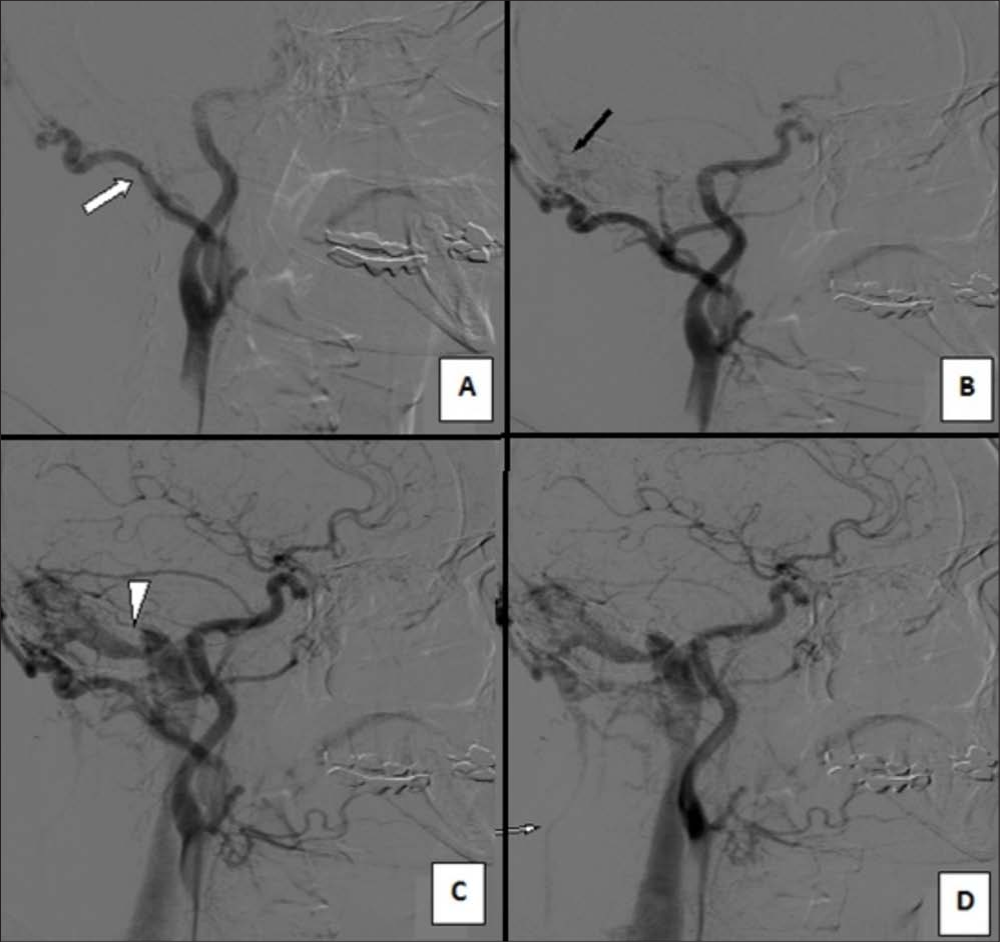
On digital subtraction angiography (DSA), (a) the enlarged right occipital (feeding) artery (white arrow), (b) the location of the fistula in the suboccipital-mastoid region (black arrow), (c) the typical drainage of DAVF to the right transverse-sigmoid sinus (white arrowhead), and (d) auxiliary/alternative venous drainage path to the right brachiocephalic vein (thin white arrow) were determined.

In addition to these findings, a subcutaneous vascular structure that showed high flow rate (128 cm/sec) and low resistance (RI = 0.48) was demonstrated in the right suboccipital-mastoid region (Figure 2). Considering all these vascular abnormalities, a CT angiography (CTA) was planned. CTA revealed a Cognard type I DAVF in the right mastoid region; its feeding artery was the occipital branch of the right ECA, and its venous drainage was to the ipsilateral transverse-sigmoid sinus. Interestingly, we noted an additional extracranial venous drainage path to the right brachiocephalic vein passing between the trapezius and levator scapulae muscles (Figure 3). Digital subtraction angiography (DSA) confirmed the diagnosis and the findings (Figure 4). The fistula was successfully treated by coil and cyano-acrylat embolization via a combined transarterial-transvenous approach.

## Discussion

Systemic diseases such as anemia, thyrotoxicosis, and valvular heart disease may cause pulsatile tinnitus since they may cause hemodynamic changes in the vascular system. Regarding the local causes of pulsatile tinnitus, DAVFs are considered among the most common causes, especially in patients with normal otoscopic examination [1,5].

Although the underlying causes for the development of DAVFs are not exactly known, it is generally believed that they are acquired due to conditions such as venoocclusive disease (venous sinus thrombosis, stenosis) trauma, surgery, infections, and certain disease entities (i.e., Osler-Weber-Rendue disease) [6]. Although various grading systems have been proposed to classify DVAFs [7,8,9], the Cognard system is the most detailed and elaborates on the direction of flow and the presence or absence of cortical venous recruitment (Table 1) [10].

**Table 1:** Cognard classification of DAVFs [10].

Type	Venous Drainage	Flow Pattern in Sinus	Cortical Venous Drainage

**Benign**			
**I**	Dural sinus	Antegrade	No
**IIa**	Dural sinus	Retrograde	No
**Aggressive**			
**IIb**	Dural sinus	Antegrade	Yes
**IIa+b**	Dural sinus	Retrograde	Yes
**III**	Cortical vein		Yes
**IV**	Cortical vein		Yes + with venous ectasia
**V**	Spinal perimedullary vein		Yes

CDS can play a role as an initial screening and follow-up tool, considering the convenience, noninvasiveness, low cost, and reproducibility of its results [11]. In our patient, CDS revealed hemodynamic differences between the right and left CCAs and ECAs and high flow rate and low resistance consistent with a DAVF in the right suboccipital-mastoid region. We believe that an ipsilateral increased blood flow and low resistive indices of carotid arteries should be considered an important finding that may be associated with a DAVF in patients with pulsatile tinnitus. Thus, CDS constitutes a very useful diagnostic tool in this clinical setting. Nevertheless, it should be noted that cranial CT and CTA, cranial MR and MR angiography, and DSA generally have crucial roles in the diagnostic workup of patients with tinnitus and specifically for patients with DAVFs. DSA, with its transarterial or transvenous intervention capabilities, is a therapeutic tool as well.

To exactly define the feeding arteries and the drainage ways of a DAVF is of crucial importance for the planning of a proper therapy. The unique finding we encountered in our patient was the venous drainage pattern of the DAVF. Besides the typical (Cognard type I) venous drainage of the ipsilateral sinus, we encountered a second drainage to the ipsilateral brachiocephalic vein. To the best of our knowledge, a drainage path like this has not been described before and is therefore not included in the classification-grading systems. We speculate that this condition may have been the consequence of high-flow DAVF involving a large area.In conclusion, in patients with pulsatile tinnitus, hemodynamic abnormalities encountered with CDS may relate to a DAVF. It should be remembered that DAVFs may have alternative or auxiliary drainage patterns. A thorough diagnostic workup that includes CTA, MRA, or DSA is needed to establish definite proper diagnosis and to guide the therapy.

## Competing Interests

The authors declare that they have no competing interests.
